# Use of Artificial Intelligence in Peer Review Among Top 100 Medical Journals

**DOI:** 10.1001/jamanetworkopen.2024.48609

**Published:** 2024-12-03

**Authors:** Zhi-Qiang Li, Hui-Lin Xu, Hui-Juan Cao, Zhao-Lan Liu, Yu-Tong Fei, Jian-Ping Liu

**Affiliations:** 1 Centre for Evidence-based Chinese Medicine, Beijing University of Chinese Medicine, Beijing, China; 2 School of Economics, Anhui University, Anhui, Hefei, China; 3 The National Research Center in Complementary and Alternative Medicine (NAFKAM), Department of Community Medicine, Faculty of Health Science, UiT the Arctic University of Norway, Tromsø, Norway

## Abstract

This cross-sectional study of 100 top medical journals examines policies for use of artificial intelligence (AI) and generative AI in peer review.

## Introduction

The rapid growth of medical research publishing and preprint servers appears to be straining the peer review process, potentially causing a shortage of qualified reviewers and slower reviews. Repeated reviews of rejected manuscripts likely increase costs, raising concerns about the system’s efficiency and fairness. Innovative solutions are urgently needed.^1^ Recent advancements in artificial intelligence (AI), particularly generative AI (GenAI), offer potential for enhancing peer review,^2^ but its integration into this workflow varies by journals policy. A comprehensive survey of medical journals’ guidance is needed to understand attitudes toward AI-assisted peer review and the reasons.

## Methods

Using data from Scimago.org,^3^ we selected the top 100 medical journals for the guidance on the use of AI in peer review, which differed from the previous instructions to authors.^4^ We searched journals’ website for AI-related policies on June 30, 2024, and August 10, 2024. If a journal lacked explicit AI guidance but recommended or linked to the publisher’s guidance, we adopted the latter as a substitute. Data extraction focused on specific AI guidance in peer review (eMethods in Supplement 1). Data were analyzed using descriptive statistics and presented as frequencies and percentages. This report follows the Strengthening the Reporting of Observational Studies in Epidemiology (STROBE) reporting guideline.

## Results

**Figure 1.**
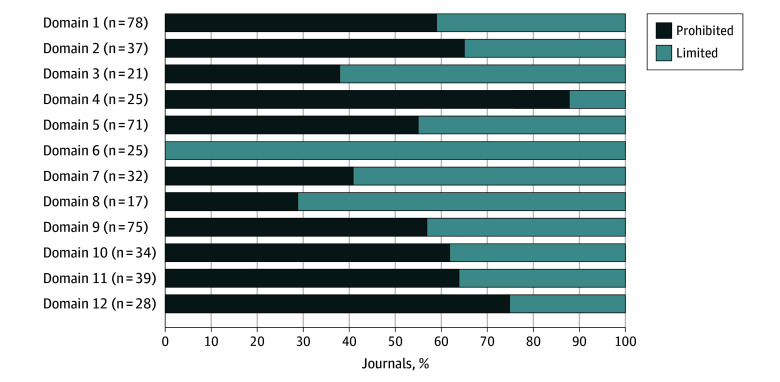
Guideline Domains for Prohibited or Limited Use Artificial Intelligence (AI) During Peer Review Process Domains defined as follows: 1, mention of AI or generative AI (GenAI); 2, mention of chatbot; 3, mention of large language models; 4, mention of other AI tools; 5, uploading any part of the manuscript to AI or GenAI is prohibited; 6, disclose AI or GenAI tools and contents used in peer review report; 7, journal guidance links to the publisher’s website; 8, following International Committee of Medical Journal Editors or World Association of Medical Editors statements on AI or GenAI; 9, reasons for prohibited or limited use include protecting the confidentiality of manuscripts; 10, reasons for prohibited or limited use include AI may generate incorrect, incomplete, or biased information; 11, reasons for prohibited or limited use include violating proprietary and data privacy rights; 12, reasons for prohibited or limited use include the expertise of peer reviewers is invaluable and irreplaceable. Each journal cited 1 or more reasons from domains 9 to 12.

**Figure 2.**
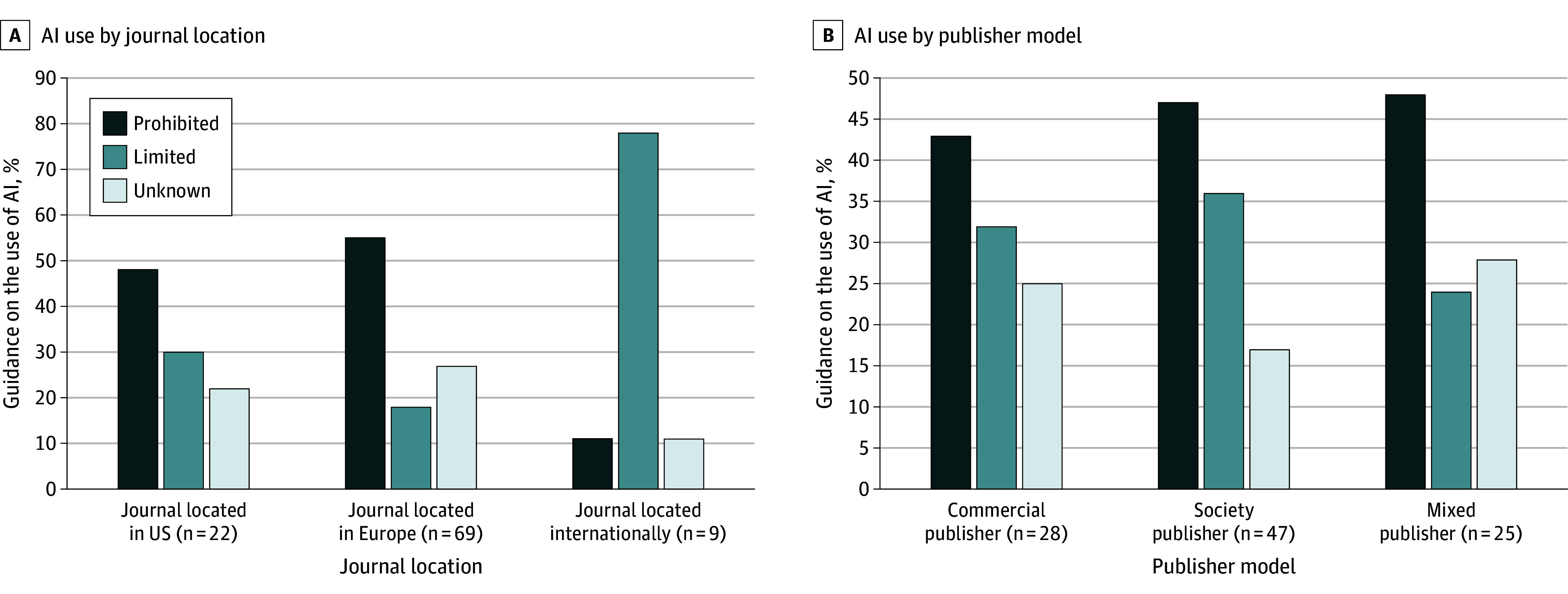
Use of Artificial Intelligence (AI) by Peer Reviewers During Peer Review Process

Overall, 78 medical journals (78%) provided guidance on use of AI in peer review. Of these provided guidance, 46 journals (59%) explicitly prohibit using AI, while 32 allow its use if confidentiality is maintained and authorship rights were respected (Figure 1). Internationally based medical journals are more likely to permit limited use than journals’ editorial located in the US or Europe, and mixed publishers had the highest proportion of prohibition on AI use (Figure 2). Notably, among the journals that provided guidance, 71 (91%) prohibited uploading manuscript-related content to AI, and 25 (32%) permitted restricted use of AI that mandated reviewers disclose in review reports. Regarding the mention of AI tools, 37 journals (47%) cite chatbots, and 21 (27%) mention large language models. In addition, 32 journals (41%) link to the publisher’s website, which had preferences in AI use—Wiley and Springer Nature favored limited use of AI, while Elsevier and Cell Press prohibited AI use. A total of 17 journals (22%) also provide links to statements from the International Committee of Medical Journal Editors or World Association of Medical Editors that permit limited use of AI (eMethods in Supplement 1), although 5 journals’ guidance contradict these statements. Variations in report proportions are observed across domains 1 to 12 in journals classified as prohibited or with limited AI use (Figure 1). Furthermore, the main reason for prohibiting or limited use of AI is confidentiality concerns (75 journals [96%]).

## Discussion

This study reveals 3 attitudes of mainstream medical journals toward AI-assisted peer review. A minority of journals (32%) allowed limited AI use, but disclosure standards varied, with critical areas like innovation, reproducibility, and reference management still underdiscussed. AI-related guidance is scattered, making it difficult for reviewers to access and understand, potentially causing misuse and confidentiality issues. But editorials could help clarify and adherence to these instructions.^5^ Despite GenAI’s potential benefits to enhance review efficiency, concerns remain about its inherent problems, which could lead to biases and confidentiality breaches.^6^ Not all reviewers are qualified in methodology and expertise, but a survey revealed that around half of AI-involved researchers positively view the editorial and review processes that used AI.^7^ Although AI is not expected to replace human peer review, its role is expected to grow as our familiarity with AI and its technical capabilities advances. Used safely and ethically, AI can increase productivity and innovation. Thus, continuous monitoring and regular assessment of AI’s impact are essential for updating guidance, thereby maintaining high-quality peer review. This study’s limitations include considering the top medical journals, possibly overlooking lower-ranked ones’ policies. Additionally, using shared publisher guidance as a proxy may overestimate the prevalence of AI guidance. Publishers like Wiley and Springer Nature preferred limited use of AI, while Elsevier and Cell Press prohibited its use. This divergence in policy may be the ultimate reason for the observed variations in guidance.

## Notes

Supplement 1.
eMethods.

Supplement 2.
Data Sharing Statement
