# Supplementary material for: Use of Artificial Intelligence in Peer Review Among Top 100 Medical Journals
Source: JAMA Netw Open. 2024 Dec 3;7(12):e2448609. doi: 10.1001/jamanetworkopen.2024.48609 (PMC11615706; doi:10.1001/jamanetworkopen.2024.48609)
Supplement: Supplement 1. — eMethods. [file jamanetwopen-e2448609-s001.pdf]

Supplemental Online Content

Li Z, Xu H, Cao H, Liu Z, Fei Y, Liu J. Use of artificial intelligence in peer review among top 100 medical journals. *JAMA Netw Open.* 2024;7(12):e2448609. doi:10.1001/jamanetworkopen.2024.48609

eMethods

This supplemental material has been provided by the authors to give readers additional information about their work.

© 2024 Li Z et al. *JAMA Network Open.*

eMethod.

Design Cross sectional study.

Setting Top 100 medical journals ranked by the Hirsch index in the 2023 Medicine category, were selected to investigate their guidelines on the use of AI in peer review.

Guidance definition Any guidance addressing the use of AI-assisted tools, such as Chatbots, ChatGPT, large language models (LLMs) , and other tools in peer review.

Definition of opinions of journal's guidance

(1)Prohibited use :When a journal explicitly states that reviewers should not upload the manuscript of authors into an externally hosted AI tool, even if it is solely for the purpose of improving language and readability, or if the journal directly prohibits.

(2)Limited use: If a journal requires the reviewer to disclose the AI tools utilized or demands transparency regarding their usage. Or reviewers must request permission from the journal prior to using AI technology to facilitate their review.

Definition of journal's editorial location

A journal's editorial location is based on where its press or publisher is located. If a journal's editorial office is situated in only one country or region, that's its editorial location. However, for journals that are co-published by multiple presses or publishers and have editorial offices in two or more different countries/regions, their editorial location should be defined as international.

Definition of publisher model

(1)Commercial publisher: A journal is considered to fall under the commercial publishers category if its copyright is held by a commercial publishing house and is

© 2024 Li Z et al. *JAMA Network Open.*

published by itself or another commercial publisher.

(2)Society publisher: A journal is classified as a society publisher if its copyright is owned by an academic or professional society and is published in-house.

(3)Mixed publisher: When a journal's copyright is held by an academic or professional society but is published and distributed by a commercial publisher. This model is characterized as a mixed publisher.

Links to ICMJE or WAME statements on using AI

(1) International Committee of Medical Journal Editors (ICMJE). See the ICMJE’s "Recommendations for the Conduct, Reporting, Editing, and Publication of Scholarly Work in Medical Journals" (http://www.icmje.org/icmje-recommendations.pdf).

(2) World Association of Medical Editors (WAME). See the WAME’s “Chatbots, Generative AI, and scholarly manuscripts: WAME recommendations on Chatbot s and Generative artificial intelligence in relation to scholarly publications” (htt ps://wame.org/page3.php?id=106).

URLs shared by some journals

(1) **Elsevier:** https://www.elsevier.com/about/policies-and-standards/the-use-of-gener ative-ai-and-ai-assisted-technologies-in-the-review-process;

(2) **Springer Nature:** https://www.nature.com/nature-portfolio/editorial-policies/pee r-review#ai-use-by-peer-reviewers;

(3) **Wiley:** https://authorservices.wiley.com/ethics-guidelines/index.html;

(4) **Cell Press:** https://www.cell.com/reviewers

(5) **JAMA Network**: https://jamanetwork.com/journals/jama/fullarticle/2807956;

© 2024 Li Z et al. *JAMA Network Open.*

(6) **Lancet series journals:** https://www.thelancet.com/peer-review;

(7) BMJ series journals: https://www.bmj.com/content/ai-use;

(8) **American Heart Association(AHA)**: https://www.ahajournals.org/for-reviewers;

(9) American Chemical Society(ACS): https://researcher-resources.acs.org/publish/peer_reviews;

(10) American Association for Cancer Research(AACR):https://aacrjournals.org/pages/editorial-process#peerrev.

Data collection time: Initial data collection from the website of journals were completed on July 30th, 2024, with an update on August 10th, 2024. No additional guidance were reported between these searches. The last search was conducted at 10.1 during the revision period.

Retrieval mode: Two reviewers (LZQ and XHL) independently collected the data, with discrepancies resolved by a third reviewer (LJP).

© 2024 Li Z et al. *JAMA Network Open.*
