# Supplementary material for: Use of Artificial Intelligence in Peer Review Among Top 100 Medical Journals
Source: JAMA Netw Open. 2024 Dec 3;7(12):e2448609. doi: 10.1001/jamanetworkopen.2024.48609 (PMC11615706; doi:10.1001/jamanetworkopen.2024.48609)
Supplement: Supplement 2. — Data Sharing Statement [file jamanetwopen-e2448609-s002.pdf]

Data Sharing Statement

Li. Guidance on Use of AI in Peer Review Among Top 100 Medical Journals. *JAMA Netw Open*. Published December 03, 2024. doi:10.1001/jamanetworkopen.2024.48609

Data
Data available: Yes
Data types: Data (not involving human participants), Data dictionary
How to access data: Data (not involving human participants),lizhiqiang@bucm.edu.cn
When available: With publication

Supporting Documents
Document types: None

Additional Information
Who can access the data: Anyone requesting the data
Types of analyses: For any purpose or for a specified purpose
Mechanisms of data availability: After approval of a proposal, or with a signed data access agreement
Any additional restrictions: NA
